# Supplementary material for: Probabilistic adaptation of language comprehension for individual speakers: evidence from neural oscillations
Source: Soc Cogn Affect Neurosci. 2025 Aug 14;20(1):nsaf085. doi: 10.1093/scan/nsaf085 (PMC12542498; doi:10.1093/scan/nsaf085)
Supplement: nsaf085_Supplementary_Data [file nsaf085_supplementary_data.docx]

**Supplementary Materials**

| **Table S1a. Experimental items used in target trials** | | |
| --- | --- | --- |
| Sentence | | English translation |
| Congruent with adult speakers but incongruent with child speakers | | |
|  | 我和我以前的**爱人**曾一起开过一家店 | My former **lover** and I used to run a store together |
|  | 我喜欢通过做**按摩**来让肌肉放松 | I like to have a **massage** to relax my muscles |
|  | 上个月我以**伴郎**的身份参加了好朋友的婚礼 | I attended my best friend’s wedding as **best man** last month |
|  | 我和我现在的**伴侣**生活在城市郊区 | My current **partner** and I live in the outskirts of the city |
|  | 我希望我买的**彩票**能够中大奖 | I hope that my **lottery ticket** will win the jackpot |
|  | 我过去有一些包括**抽烟**在内的不健康的生活习惯 | I have had some unhealthy habits like **smoking** in the past |
|  | 我参加过很多次**慈善**活动了 | I’ve participated in many **charity** events before |
|  | 我把我**存款**的一部分留作应急开支 | I’m putting some of my **savings** aside for emergencies |
|  | 我每个月都有很多**贷款**要还包括房贷和车贷 | I have a lot of **mortgage** payments every month including a mortgage and a car loan |
|  | 我以前因为**赌博**欠下过很多债 | I used to **gamble** and have a lot of debt |
|  | 我现在已经很少去**赌场**这类场所了 | I rarely go to **casinos** anymore |
|  | 我在刚刚**分手**后的那段时间里频繁感到焦虑和疲倦 | I had a **breakup** and felt anxious and tired all the time |
|  | 有时候我觉得自己为了**工作**牺牲了大量私人时间 | I sometimes feel like my **work** sacrifices a lot of my personal time |
|  | 他们叫我买的**股票**都还不错我赚了很多钱 | I bought all the **stocks** they told me to buy and I made a lot of money |
|  | 这段时间我被各种**官司**搞得焦头烂额 | I’ve been caught up in a lot of **lawsuits** in the meantime |
|  | 我会认真读**合同**上的每一个条款不会轻易签字 | I read the **contracts** carefully and don’t sign them lightly |
|  | 我最近在研究一些**基金**的派息政策 | I’ve been studying some **funds’** dividend policies |
|  | 我每天要做的**家务**特别多 | I’m doing a lot of **housework** every day |
|  | 我今天在改我的**简历**上花了很多时间 | I spent a lot of time on my **resume** today |
|  | 我已经坚持**健身**好几年了 | I’ve been going to the **gym** for years |
|  | 他们都夸我的**接吻**技术很好 | Everyone praises my **kissing** skills |
|  | 我喜欢晚上去**酒吧**喝酒放松 | I like to go to pubs at night to **drink** and relax |
|  | 我有每天早上喝**咖啡**的习惯 | I drink **coffee** every morning |
|  | 我平时**开车**的时候很注意交通安全 | I’m always **driving** in a safe manner |
|  | 我在每一段**恋爱**中都是付出的那个人 | I am the one in every **relationship** who gives |
|  | 我觉得我一个人**旅行**最大的意义是获得心灵的平静 | I think I **travel** alone most to gain peace of mind |
|  | 我现在的**收入**还算可观 | I’m making a good **income** right now |
|  | 我在每次**谈判**前都会做充分的准备 | I prepare myself well before every **negotiation** I have |
|  | 我现在离**退休**还有很多年 | I still have a few years left before I **retire** from my job |
|  | 我最近在想怎么把我的**遗产**分配给我的家人 | I’ve been trying to figure out how to divide my **inheritance** among my family |
|  | | |
| Congruent with child speakers but incongruent with adult speakers | | |
|  | 我希望自己能像**超人**一样在天上飞 | I wish I could be **Superman** and fly in the sky |
|  | 我总是因为**闯祸**而遭到我爸的打骂 | I was always scolded for **getting into trouble** by my father |
|  | 我在准备去**春游**要带的零食 | I was preparing for the **spring break** snacks I’d bring |
|  | 我常常因为跟别人**打闹**而被我爸骂 | I always got yelled at for **messing around** with other people by my dad |
|  | 我们在楼下玩**弹弓**的时候特别开心 | We had so much fun playing **slingshot** downstairs |
|  | 我经常因为**捣蛋**而惹我妈生气 | I always **make trouble** and get my mom mad |
|  | 我经常在家附近的公园里玩**滑梯**和蹦蹦床 | I often **play on the slide** and trampoline at the park near my home |
|  | 我喜欢用**积木**搭好看的房子 | I like to use **legos** to build nice houses |
|  | 我老是因为各种问题被我**家长**领回家 | I was always taken home by my **mom and dad** for all sorts of problems |
|  | 我最喜欢看**卡通**和读故事 | My favorite things are watching **cartoons** and reading stories |
|  | 他们经常夸我又**可爱**又活泼 | They used to praise me for being **cute** and lively |
|  | 我最近开始不用**奶瓶**喝奶了 | I’ve recently quit the **milk bottle** and started drinking without it |
|  | 他们送了我一套**拼图**作为礼物 | They gave me a set of **jigsaw puzzles** as a gift |
|  | 我正在通过学**拼音**来认汉字 | I am learning **Pinyin** to recognize Chinese characters |
|  | 我最近学会**骑车**之后觉得太有趣了 | I recently learned how to **ride** a bike and it’s so much fun |
|  | 我从来不会给陌生的**叔叔**开门让他进到家里来 | I never opened the door for a **strange uncle** to come into the house |
|  | 我们在一起玩**水枪**的时候特别开心 | We had a lot of fun playing **water pistols** together |
|  | 我最近学**算数**有很大进步 | I’ve made great progress in **counting** lately |
|  | 我喜欢把从家里带来的**糖果**跟大家分着吃 | I like to share the **candies** I brought from home with everyone |
|  | 我是个很**淘气**的人经常在家里搞恶作剧 | I’m a very **naughty** person who often plays pranks at home |
|  | 我因为太**调皮**老是被我妈批评 | I was always accused of being **peevish** by my mom |
|  | 他们都说我要是再**听话**一些就好了 | They say I should have **behaved** better |
|  | 我喜欢听别人讲**童话**里的英雄故事 | I like to listen to **fairy tales** about heroes |
|  | 我经常因为乱**涂鸦**而被我妈骂 | I often get scolded for **graffiti** by my mom |
|  | 他把我的**玩具**抢走了 | He took my **toys** away from me |
|  | 我最喜欢的**玩偶**是一只白色的小猫 | My favorite **doll** is a white kitten |
|  | 我总是因为**顽皮**而被我妈训 | I was always scolded for being a **brat** by my mom |
|  | 我每天晚上必须得抱着我的**小熊**才能睡着 | I have to hold my **teddy bear** every night to fall asleep |
|  | 我最近正在学**写字**和画画 | I’m learning to **write the alphabet** and to draw |
|  | 我得把我的**作业**写完才能出去玩儿 | I have to finish my **homework** before I can go out and play |
|  | | |
| Congruent with female speakers but incongruent with male speakers | | |
|  | 我喜欢和他们一起聊八**卦**和有意思的事 | I like to **gossip** and talk about interesting things with them |
|  | 我睡觉的时候习惯把我的**辫子**解开以免不舒服 | I used to undo my **braids** when I slept to avoid discomfort |
|  | 在夏天我会把我的**长发**剪短一些 | In the summer I cut my **long hair** short |
|  | 我喜欢穿着**长裙**站在微风中的那种飘逸的感觉 | I love the feeling of wearing a **dress** and standing in the breeze |
|  | 我一向都是给人一种很**端庄**的印象 | I’ve always had a very **demure** look |
|  | 我的新**耳环**是纯金的 | My new **earrings** are solid gold |
|  | 我喜欢一切**粉色**的东西包括衣服鞋子和包包 | I love all things **pink** including clothes shoes and handbags |
|  | 我偶尔会展现出特别**风骚**的一面来惊艳众人 | I can occasionally look particularly **flirty** to impress people |
|  | 见客人前我都会**化妆**确保形象完美 | I always wear **makeup** before I meet a client to make sure I look perfect |
|  | 我放松的方式是和**姐妹**们去购物 | I go shopping with the **girls** as a way to relax |
|  | 我过生日朋友们经常送我**口红**作为礼物 | My friends always give me **lipstick** as a gift for my birthday |
|  | 他们说我走路的姿势特别**曼妙**引人侧目 | They say my walk is so **graceful** that it draws people’s attention |
|  | 我每个月都要去做一次**美发**和面部护理 | I get a **hairdressing** and facial once a month |
|  | 这周末我要先去做**美甲**然后理发 | This weekend I’m getting a **manicure** and a haircut |
|  | 很多人都因为我的**美貌**而默默关注着我 | My **beauty** has attracted a lot of attention |
|  | 我预约了明天去做**美容**和按摩 | I made an appointment for a **facial** and massage tomorrow |
|  | 有时候我会展现我很**俏皮**的一面 | Sometimes I show my **flirty** side |
|  | 我经常穿**裙子**出门逛街 | I often wear **skirts** when I go out shopping |
|  | 我打算攒钱买一个名牌**手袋**来奖励自己 | I’m going to save up and buy a designer **handbag** to reward myself |
|  | 我喜欢戴**手镯**来装饰我的手腕 | I like to wear **bracelets** to decorate my wrists |
|  | 我有时会戴一条**丝巾**来提升我的气质 | I sometimes wear a **silk scarf** to enhance my look |
|  | 我喜欢穿黑色的**丝袜**来让腿显得修长 | I like to wear black **stockings** to make my legs look slim |
|  | 我从小就喜欢通过**跳舞**来让自己放松 | Since I was a kid I’ve always loved **dancing** to relax myself |
|  | 我有时会戴**头巾**来修饰我发型的轮廓 | I sometimes wear a **headscarf** to contour my hair |
|  | 出门的时候我一般都会戴**头饰**来装点我的气质 | When I go out I usually wear a **headpiece** to make me look good |
|  | 我在大家面前一直是很**温柔**的形象 | I’ve always been a very **sweet** person in front of everyone |
|  | 我有时候会展现出很**妩媚**的一面 | I can be quite **flirtatious** at times |
|  | 这周末我会戴我最喜欢的**项链**参加舞会 | I’m wearing my favorite **necklace** to prom this weekend |
|  | 我最近在学**绣花**和织围脖 | I’ve been learning to **embroider** and knit scarves |
|  | 大家都说我的站姿**优美**很有气质 | They say my standing posture is **graceful** and elegant |
|  | | |
| Congruent with male speakers but incongruent with female speakers | | |
|  | 我早年在酒店当**保安**的时候过得很辛苦 | I had a hard time working as a hotel **security guard** in my early years |
|  | 我过去当**保镖**的时候遇到过一些紧急事件 | I’ve had some emergencies as a **bodyguard** in the past |
|  | 我每周末都在健身房练习**搏击**和其他项目 | I’m in the gym every weekend practicing **sparring** and other sports |
|  | 我小时候经常**打架**被老师叫家长 | When I was a kid I used to **get into fights** and my teacher called my parents |
|  | 我小时候有一次因为**斗殴**而被学校给了处分 | I was once disciplined for a **brawl** when I was small |
|  | 我特别喜欢进行**格斗**类的运动 | I’m a big fan of **combat** sports |
|  | 我早年留**光头**的时候对自己的造型特别自信 | In my early days I had a **bald head** and I was very confident in the way I looked |
|  | 我经常练习**举重**来增强肌肉力量 | I regularly practiced **weight lifting** to build up my muscle strength |
|  | 我非常向往成为一名**军人**保家卫国 | I really wanted to be a **soldier** to protect my country |
|  | 我特别爱和别人聊**军事**和政治 | I love talking to people about the **military** and politics |
|  | 我曾经以开**卡车**拉货为生 | I used to work as a **truck** driver for a living |
|  | 我近几年的**扣篮**技术不如以前了 | I’m no longer as good at **dunking** as I used to be |
|  | 我喜欢和朋友们在公园打**篮球**和排球 | I like to play **basketball** and volleyball with my friends at the park |
|  | 过节的时候我经常收到**领带**这样的礼物 | I often get gifts like **ties** for the holidays |
|  | 我参加重要宴会的时候会戴**领结**穿正装 | I wear a **bow tie** and a formal dress when I go to important parties |
|  | 我不喜欢有的人形容我的时候用**流氓**这个词 | I don’t like to be described as a **thug** by others |
|  | 我以前在很多地方做过**门卫**的工作 | I used to work as a **gatekeeper** in many places |
|  | 我最喜欢看**枪战**类的电影因为感觉很刺激 | I like to watch **gunfighting** movies because they’re exciting |
|  | 我最喜欢的运动是**拳击**和足球 | My favorite sports are **boxing** and soccer |
|  | 我非常喜欢**赛车**运动希望有一天可以亲自参加比赛 | I love **motor sports** and hope to race one day |
|  | 我不喜欢有的人叫我**色狼**来开玩笑 | I don’t like it when people call me a **pervert** as a joke |
|  | 在公众场合我会展示**绅士**的一面 | I will show a **gentleman’s** side in public |
|  | 我最近正在为下个月参加**摔跤**比赛积极做准备 | I’m preparing for a **wrestling** match next month |
|  | 他们总夸我**帅气**得像明星 | They always say I’m as **handsome** as a movie star |
|  | 我曾经作为一名**司机**往返各地送货 | I used to work as a **driver** making deliveries all over the city |
|  | 在工作单位我一般都是穿**西服**和衬衫 | I usually wear a **suit** and shirt at work |
|  | 这周末我约了好**兄弟**一起吃饭 | This weekend I’m meeting my **buddy** for dinner |
|  | 朋友经常跟我说我给人一种很**阳刚**的感觉 | My friends always tell me that I look very **masculine** to them |
|  | 他们经常夸我又**英俊**又有格调 | They always tell me I look **smart** and have style |
|  | 我小时候的梦想是当一名**战士**冲锋陷阵 | I dreamed of being a **soldier** when I was a kid |
| The critical word in a sentence is marked. | | |

| **Table S1b. Experimental items used in filler trials** | | |
| --- | --- | --- |
| Sentence | | English translation |
| Congruent with adult speakers but incongruent with child speakers | | |
|  | 我买了各种**保险**包括医疗险和意外险 | I have all kinds of **insurance** including medical and accident insurance |
|  | 我有时候会在**茶馆**和老朋友喝茶聊天 | I sometimes go to **tea houses** to drink tea and chat with old friends |
|  | 下个月我要**出差**去外地工作一个星期 | I’m going on a **business trip** next month for a week’s work overseas |
|  | 我打算今年跟朋友一起**创业**做外贸生意 | I plan to **launch a business** in foreign trade with a friend this year |
|  | 我在为后天的**答辩**做准备 | I’m preparing for my **defense** the day after tomorrow |
|  | 我已经**订婚**一年了 | I’ve been **engaged** for a year |
|  | 我借过一次**高利贷**因为有紧急情况需要资金周转 | I took out a **loan** from a loan shark once because I had an emergency and needed the money |
|  | 我打算早点**结婚**让我父母放心 | I’m trying to **get married** early to reassure my parents |
|  | 我已经尝试**戒烟**一段时间了但是没有成功 | I’ve been trying to **quit smoking** for a while now but I haven’t succeeded |
|  | 我还想在**经济学**方面继续深耕一下 | I’d like to get into **economics** a bit more |
|  | 我觉得获得一些**就业**方面的指导是很有帮助的 | I think getting some **career** guidance would be helpful |
|  | 我最近想通过**理财**赚一点钱 | I’ve been trying to **manage my finances** to make a little money lately |
|  | 我每年夏天要去海边**疗养**半个月 | I take a **retreat** to the beach for half a month every summer |
|  | 我昨天和大家讨论**伦理**问题有很多收获 | I learned a lot about **ethics** from the discussion yesterday |
|  | 我最近正在考虑**买房**的事情 | I’ve been thinking about **buying a house** lately |
|  | 我最喜欢喝**啤酒**吃烧烤了 | My favorite thing to do is to drink **beer** and eat barbecue |
|  | 我有时候会通过**染发**来让自己换个心情 | I sometimes use **hair dyes** to change my mood |
|  | 我喜欢做**桑拿**来让身体排毒 | I like to do **sauna** to detoxify my body |
|  | 我经常穿**商务**风格的衣服 | I often wear **business style** clothes |
|  | 我喜欢做**水疗**来放松身体 | I like to go to the **spa** to relax my body |
|  | 我每个月要做一次**烫发**和头皮护理 | I get a **perm** and scalp treatment once a month |
|  | 这个假期我要去**跳伞**和蹦极 | I’m going **skydiving** and bungee jumping on this vacation |
|  | 我打算做一些**投资**来实现财富增值 | I’m going to **invest** some money to grow my wealth |
|  | 我有时候会去做**推拿**治疗腰痛 | I sometimes go for **massage** for my back pain |
|  | 我每天晚上都会先喝一杯**威士忌**再上床睡觉 | I drink a **scotch** every night before I go to bed |
|  | 我一般都是用**信用卡**消费因为有积分 | I usually use my **credit card** to pay for my purchases because I earn points |
|  | 我今天晚上要带一瓶**洋酒**去参加朋友的派对 | I’m taking a bottle of **liquor** to a friend’s party tonight |
|  | 他们说我作为**养生**专家给的建议都很实用 | They say my advice as a **health** expert is very useful |
|  | 我有时候去**夜总会**一待就是一整夜 | I sometimes go to **nightclubs** and stay all night |
|  | 我每周都有几天需要**应酬**到很晚 | I have to **socialize** late a couple days a week |
|  | | |
| Congruent with child speakers but incongruent with adult speakers | | |
|  | 我希望自己能像**奥特曼**一样打败怪兽拯救人类 | I wish I could be like **Ultraman** and defeat monsters to save mankind |
|  | 他们每次来看我的时候都会给我带一支**棒棒糖**给我吃 | They bring me a **lollipop** to eat every time they visit me |
|  | 我会在地上**打滚**让他们给我买零食 | I’d **roll** on the ground to get them to buy me snacks |
|  | 我最喜欢看的**动画**连续剧是在每周二播出 | My favorite **animation** series is on Tuesdays |
|  | 我今年过**儿童节**的方式是去吃麦当劳 | My way to celebrate **Children’s Day** this year is to go to McDonald’s |
|  | 我洗完澡之后会有人帮我用**痱子粉**来涂全身 | After I take a bath someone will help me apply **baby powder** all over my body |
|  | 我每周都要去**辅导班**学写字 | I go to a **tutorial class** to learn how to write every week |
|  | 有时候他们会夸我**乖巧**有礼貌 | Sometimes they praise me for being **good** and polite |
|  | 我们经常在一起玩**过家家**的游戏 | We often **play house** together |
|  | 我再怎么**哭鼻子**他们也不会答应我过分的要求 | No matter how much I **cried** they wouldn’t give in to my excessive demands |
|  | 他们总害怕我会在飞机上**哭闹**打扰别人 | They were always afraid I’d **cry** on the plane and disturb others |
|  | 我刚拿到**零花钱**一小时不到就全都花掉了 | I just got my **pocket money** and spent it all in less than an hour |
|  | 我喜欢看**漫画**虽然我有很多字看不懂 | I like reading **comics** even though I can’t understand a lot of the words |
|  | 我最爱在院子里**玩泥巴**捏各种各样的造型 | I love to **play with mud** in the yard and make all kinds of shapes |
|  | 我特别喜欢在广场上玩**吹泡泡**的游戏 | I especially like **blowing bubbles** in the square |
|  | 上周我玩**跷跷板**的时候把腿摔伤了 | Last week I was playing on the **see-saw** and hurt my leg |
|  | 我经常在楼下玩**秋千**玩到很晚 | I often stay up late playing on the **swing** downstairs |
|  | 我周末参加**少年宫**的围棋班 | I go to a **youth centre’s** Go class on weekends |
|  | 他们让我改掉**吃手指**的坏习惯 | They told me to stop **eating my fingers** as a bad habit |
|  | 他们经常说我**挑食**老是不吃青椒和胡萝卜 | They often say I’m a **picky eater** never eating peppers or carrots |
|  | 我回家的时候经常边哼着**童谣**边走路 | I often hum **nursery rhymes** as I walk home |
|  | 我今年收到的生日礼物是一件**童装**和一双运动鞋 | For my birthday this year I got a **children’s dress** and a pair of sneakers |
|  | 现在他们还经常给我**喂奶**让我慢慢喝 | Now they still **feed me milk** and let me drink it slowly |
|  | 我经常在海边**嬉戏**玩耍特别开心 | I had a lot of fun **splashing around** on the beach |
|  | 我喜欢用**橡皮泥**来捏各种动物造型 | I like to use **play dough** to make all kinds of animals |
|  | 我把我所有的**压岁钱**都攒下来打算买一辆玩具车 | I'm saving up all the **red envelope money** for a toy car |
|  | 有人送了我一个**洋娃娃**作为礼物 | Someone gave me a **doll** as a gift |
|  | 我最喜欢去的**游乐场**这周末停业了 | My favorite **playground** is closed this weekend |
|  | 我特别爱玩**游戏机**每天晚上都要玩 | I love playing **video games** every night |
|  | 我上次学**游泳**的时候被晒伤了 | I got sunburned when I learned to **swim** last time |
|  | | |
| Congruent with female speakers but incongruent with male speakers | | |
|  | 我希望自己能像**芭蕾舞**演员一样体态优雅 | I wish I could be **balletically** graceful |
|  | 我喜欢穿**百褶裙**的那种轻松舒适的感觉 | I love wearing **pleated skirts** for the ease and comfort |
|  | 我曾经做过一段时间的**保姆**照顾小孩 | I worked as a **nanny** for a while taking care of children |
|  | 我很享受在沙滩上穿**比基尼**的感觉 | I love wearing a **bikini** on the beach |
|  | 我有时候会穿**超短裙**出门逛街 | I sometimes wear **miniskirts** when I go out shopping |
|  | 我打算穿浅色的**吊带**来搭配我的新鞋 | I’m going to wear light-colored **camisoles** with my new shoes |
|  | 我喜欢戴**发卡**来装饰我的头发 | I like to wear **hairpins** to decorate my hair |
|  | 我每次穿**高跟鞋**走在街上都觉得特别自信 | I feel confident in my **high heels** every time I walk down the street |
|  | 我最喜欢**蝴蝶结**造型的配饰 | I love to wear **bows** as my favorite accessory |
|  | 从小我父母就期待我能成为一名**护士**在医院工作 | Since I was a little girl my parents wanted me to be a **nurse** and work in a hospital |
|  | 我一直都在参加**健美操**培训课 | I’ve been taking **fitness dance** classes for a long time |
|  | 朋友们经常会说我很**娇气**但我一点都不介意 | My friends often say I’m **dainty** but I don’t mind |
|  | 我虽然看起来有点**娇羞**但其实是很外向的人 | I may look a bit **bashful** but I’m actually quite outgoing |
|  | 我的新**连衣裙**是上周刚买的 | I just bought my new **dress** last week |
|  | 我真希望自己能变成**麦当娜**那样迷人的人 | I wish I could be as glamorous as **Madonna** herself |
|  | 我很适合当一名**秘书**因为我细致有耐心 | I’m good at being a **secretary** because I’m meticulous and patient |
|  | 他们经常夸我长得很**漂亮**秀色可餐 | They always tell me I’m **pretty** and I look good |
|  | 我每周都会上几节**普拉提**课来保持身材 | I take **Pilates** classes a couple times a week to stay in shape |
|  | 我有时候喜欢穿**旗袍**出门因为可以凸显身材 | Sometimes I like to wear a **cheongsam** to go out because it emphasizes my figure |
|  | 有人说我的气质**柔美**得像一朵樱花 | I’ve been told I’m as **delicate** as a cherry blossom |
|  | 我喜欢用**头绳**把头发都扎起来 | I like to use **hairbands** to tie up my hair |
|  | 我下周要穿**晚礼服**去参加一场重要晚宴 | I’m wearing a **gown** to an important dinner next week |
|  | 他们说我说话的语气**温婉**动听让人感觉如沐春风 | They say I speak in a **melodious** tone that makes people feel like they’re in the breeze |
|  | 我从小就希望自己能像**舞蹈家**那样在台上翩翩起舞 | Since I was a little girl I’ve always wanted to be like a **dancer** on stage |
|  | 我很高兴有人能夸我**贤惠**和能干 | I’m happy to be complimented for being so **virtuous** and capable |
|  | 我有时会展现出我很**性感**的一面 | I sometimes show my **sexy** side |
|  | 我的一头**秀发**经常成为众人目光的焦点 | My **silky hair** is always the center of attention |
|  | 大家觉得我拍照的姿势有一种很**妖娆**的感觉 | People think I have a **sultry** look in my poses |
|  | 他们说我的眼神里总有一种**妖艳**的感觉 | They say there’s always a **seductive** look in my eyes |
|  | 我平时的一个爱好是**种花**和修剪盆栽 | One of my hobbies is **planting flowers** and trimming potted plants |
|  | | |
| Congruent with male speakers but incongruent with female speakers | | |
|  | 他们总说我性格**霸道**爱发号施令 | They say I’m **bossy** and always give orders |
|  | 他们都觉得我的举手投足很**霸气**非常有魅力 | They think I’m very **dominant** and charming |
|  | 我年轻的时候曾经作为**搬运工**在码头装卸货物 | When I was young I used to work as a **porter** loading goods at the docks |
|  | 我曾经报名**参军**但是落选了 | I signed up for the **military** but didn’t make the cut |
|  | 我想先上一些网课然后兼职当**程序员**来挣外快 | I’m gonna take some online classes and work part-time as a **programmer** to make some extra money |
|  | 我以前常年留**寸头**是因为不需要打理 | I used to have a **buzz cut** because I didn’t need to take care of it |
|  | 我周末去**钓鱼**在湖边一呆就是一整天 | I would go **fishing** on weekends and spend the whole day at the lake |
|  | 他们说我很有**风度**举止得体 | They say I’m very **debonair** and well mannered |
|  | 我曾经参加**橄榄球**比赛并且带着球队赢得了冠军 | I played **rugby** and won the championship with my team |
|  | 我经常给人一种很**豪放**的感觉 | I’ve always had a very **bold** look about me |
|  | 我经常在海边骑**机车**带我爱人兜风 | I often ride my **motorcycle** on the beach and take my lover for a ride |
|  | 我以后想当一名**机械师**修理各种精密设备 | I want to be a **mechanic** who repairs all kinds of precision equipment |
|  | 我上中学时的理想是当一名**机长**驾驶飞机在空中翱翔 | My dream when I was in high school was to be a **captain** and fly airplanes in the sky |
|  | 我以前的理想之一是成为一名**警察**抓捕犯罪嫌疑人 | One of my former dreams was to become a **police officer** and arrest suspects |
|  | 夏天热的时候我一般都是穿**裤衩**出门凉快又舒服 | I usually wear **boxers** when it’s hot in the summer to stay cool and comfortable |
|  | 我希望有一天能成为像**刘德华**那样有魅力的人 | I hope to be as attractive as **Andy Lau** someday |
|  | 结婚的时候我穿的是定制的**马褂**很有东方韵味 | When I got married I wore a custom-made **waistcoat** with an oriental flavor |
|  | 当我骑着**摩托车**在路上飞驰的时候我感到自由和快乐 | I feel free and happy on my **motorcycle** speeding down the road |
|  | 我曾经因为**嫖娼**被警察抓过一次 | I was caught for **soliciting prostitutes** once by the police |
|  | 在所有运动里我最擅长的是**散打**和柔道 | Of all the sports I’m best at **Shotokan** and Judo |
|  | 公司里面大家一般都叫我**师傅**很少叫我的名字 | Inside the company people usually call me **master** but seldom call me by my name |
|  | 我在一些情绪激动的情况下会突然**兽性**大发吓到别人 | When I am in an emotional state I may suddenly have an **animalistic** outburst and scare others |
|  | 我曾经梦想成为一名**特种兵**执行高危任务 | I used to dream of serving for the **Special Forces** on high-risk missions |
|  | 他们都说我给人一种很有**威严**的感觉 | They say I give off an **intimidating** vibe |
|  | 我从小就练习**武术**所以基本功非常扎实 | I’ve practiced **martial arts** since I was a child so my basic skills are very solid |
|  | 我吃完晚饭喜欢下楼和邻居**下棋**或者打牌 | After dinner I like to go and **play chess** or cards with my neighbors |
|  | 我很擅长自己**修车**不需要别人帮忙 | I’m good at car **repairs** and don’t need help |
|  | 特别正式的活动我会穿**燕尾服**出席参加 | I wear a **tuxedo** to special events |
|  | 大家觉得我的外形**硬朗**很好看 | People think I look **tough** and handsome |
|  | 我这周末会作为参赛选手参加**足球**友谊赛 | I’ll be playing in a friendly **soccer** match this weekend |
|  | | |
| Gender-neutral | | |
|  | 我喜欢看喜剧 | I like to watch comedy |
|  | 我的早饭里一般都有水果和牛奶 | I usually have fruit and milk with my morning meal |
|  | 我的一个人生理想是环游世界 | One of my ambitions in life is to travel around the world |
|  | 我经常在家附近的公园里散步 | I often walk in the park near my home |
|  | 我每天都在手机上刷短视频 | I watch short videos on my smartphone every day |
|  | 我有时候会和朋友去听演唱会 | I sometimes go to concerts with my friends |
|  | 我经常在网上看电视剧 | I often watch TV dramas online |
|  | 我经常用手机看网络小说 | I often read online novels on my smartphone |
|  | 我最近的睡眠质量还算不错 | I have been sleeping well lately |
|  | 我喜欢吃比较清淡的饭菜 | I like to eat light meals |
|  | 我在早上会比在晚上更有精力一些 | I feel more energetic in the morning than in the evening |
|  | 我所住的这个小区人很多 | The neighborhood I live in is very crowded |
|  | 我会通过多吃蔬菜和水果来补充维生素 | I take vitamins by eating more vegetables and fruits |
|  | 今年冬天我要去北方泡露天温泉 | This winter I’m going to the north to take a dip in an open-air hot spring |
|  | 我喜欢听流行音乐 | I like to listen to pop music |
|  | 我很欣赏有创新思维的人 | I appreciate people who think creatively |
|  | 我经常去亲戚家串门 | I often visit my family members |
|  | 我觉得在困难面前保持乐观特别重要 | I think it’s important to be optimistic in the face of difficulties |
|  | 我经常在楼下的面包店里买面包 | I often pick up bread from the bakery downstairs |
|  | 我喜欢认识新朋友 | I like to meet new people |
|  | 我喜欢听电台的音乐节目 | I like listening to music programs on the radio |
|  | 我有时会和朋友去爬山 | I sometimes go hiking with friends |
|  | 我会唱很多流行歌 | I can sing a lot of pop songs |
|  | 我喜欢到世界各地旅游 | I like traveling around the world |
|  | 我经常和朋友出去露营 | I often go camping with my friends |
|  | 我很少熬夜 | I rarely stay up late |
|  | 我经常在网上买东西 | I often buy things online |
|  | 我非常重视和家人的关系 | I value my relationship with my family |
|  | 我很注意在饮食上保持营养均衡 | I eat a well-balanced diet |
|  | 我经常打扫屋子 | I clean my house regularly |
|  | | |
| Age-neutral | | |
|  | 过年的时候我们家会在门上贴春联 | During the Spring Festival our family puts up Spring Festival couplets on the door |
|  | 我家楼下有一个很大的停车场 | There is a big parking lot in my neighborhood |
|  | 我以前养过一条狗 | I used to have a dog |
|  | 我每天睡觉前都会洗澡 | I take a bath every evening before I go to bed |
|  | 我家有一台苹果电脑 | I have an iMac at home |
|  | 我不喜欢用新毛巾因为会掉毛 | I don’t like to use new towels because they shed lint |
|  | 我邻居家的狗整天叫个不停 | My neighbor’s dog barks all day long |
|  | 我不喜欢用吹风机吹头发 | I don’t like using a hair dryer on my hair |
|  | 我的雨伞昨天丢了 | I lost my umbrella yesterday |
|  | 我每天早上都刷牙 | I brush my teeth every morning |
|  | 我一般中午十二点吃午饭 | I usually eat lunch at 12:00 pm |
|  | 我觉得最近天很热 | I find it very hot lately |
|  | 我觉得明天会下雨 | I feel it’s going to rain tomorrow |
|  | 我家楼下的餐厅很好吃 | The restaurant downstairs at my place has great food |
|  | 我想做一个勤劳的人 | I want to be a hard-working person |
|  | 我家楼顶每天都会有飞机飞过 | There are airplanes flying over the roof of my apartment every day |
|  | 我经常听见隔壁邻居在屋里说话 | I often hear my next door neighbor talking in the apartment |
|  | 我喜欢听别人讲笑话 | I like to hear people tell jokes |
|  | 我吃饭的时候喜欢喝饮料 | I like to have a drink when I eat |
|  | 我每天早上都会吃两个鸡蛋 | I eat two eggs every morning |
|  | 我经常一边吃饭一边看电视 | I often watch TV while eating |
|  | 我不喜欢吃太辣的东西 | I don’t like spicy food |
|  | 一到秋天我家就会刮大风 | It’s windy in my town once in the fall |
|  | 下雨天我只想在家里躺着 | I just want to lay in my bed when it’s raining |
|  | 我喜欢听节奏欢快的歌 | I like to listen to songs with a happy rhythm |
|  | 我坐长途车有时候会晕车 | I sometimes get carsick on long-distance rides |
|  | 我觉得乱发脾气是很不好的 | I think it’s bad to lose my temper |
|  | 我喜欢在阳台晒太阳 | I like to sunbathe on the balcony |
|  | 天热的时候我喜欢喝冰水 | I like drinking ice water when it’s hot |
|  | 天黑的时候我会把家里的灯都打开 | I turn on all the lights in the apartment when it’s dark |
| The critical word in a sentence is marked. For age-neutral and gender-neutral sentences, there is no critical word. | | |

| **Table S2. Channels included in amplitude analyses and TFR analyses** | |
| --- | --- |
| Analysis | Channel |
| Amplitude (in both Experiment 1 & 2) | AF3, AF4, AFF5h, AFF3h, AFF1h, AFF2h, AFF4h, AFF6h, F5, F3, F1, Fz, F2, F4, F6, FFC5h, FFC3h, FFC1h, FFC2h, FFC4h, FFC6h, FC5, FC3, FC1, FCz, FC2, FC4, FC6, FCC5h, FCC3h, FCC1h, FCC2h, FCC4h, FCC6h, C5, C3, C1, Cz, C2, C4, C6, CCP5h, CCP3h, CCP1h, CCP2h, CCP4h, CCP6h, CP5, CP3, CP1, CPz, CP2, CP4, CP6, CPP5h, CPP3h, CPP1h, CPP2h, CPP4h, CPP6h, P5, P3, P1, Pz, P2, P4, P6, PPO5h, PPO3h, PPO1h, PPO2h, PPO4h, PPO6h, PO3, POz, PO4 |
| TFR (high-beta cluster in Experiment 1) | AFF2h, AFF4h, F3, Fz, F2, FFC3h, FFC1h, FFC2h, FFC4h, FC3, FC1, FCz, FC2, FC4, FCC3h, FCC1h, FCC2h, FCC4h, C1, Cz, C2, C4, CCP3h, CCP1h, CCP2h, CCP4h, CCP6h, CP3, CP1, CPz, CP2, CP4, CPP5h, CPP3h, CPP1h, CPP2h, CPP4h, P3, P1, Pz, P2, PPO3h, PPO1h, PPO2h, POz, POO1 |
| TFR (theta cluster in Experiment 1) | CP3, CP5, CCP5h, TPP7h, CPP5h, CPP3h, P3 |
| TFR (high-beta cluster in Experiment 2) | C3, C1, Cz, TTP7h, CCP5h, CCP3h, CCP1h, TP7, CP5, CP3, CP1, CPz, CPP5h, CPP3h, CPP1h, P5, P3, P1, Pz, PPO5h, PPO3h, PPO1h, PPO2h, PO3, POz, POO5, POO1 |
|  | |

| **Table S3. LME models for amplitude analyses in Experiment 1** | | | | |
| --- | --- | --- | --- | --- |
| Predictor | *β* | *SE* | *t* | *p* |
| Models for main analyses |  |  |  |  |
| N400 (300-500 ms) |  |  |  |  |
| Intercept | -1.74 | 0.16 | -10.69 | < .001 |
| Congruency | 0.03 | 0.26 | 0.10 | 0.922 |
| Base rate | -0.36 | 0.23 | -1.58 | 0.114 |
| Critical word frequency | 0.27 | 0.13 | 2.05 | 0.042 |
| Congruency: Base rate | -0.53 | 0.45 | -1.18 | 0.239 |
| P600 (600-1000 ms) |  |  |  |  |
| Intercept | 0.06 | 0.22 | 0.27 | 0.789 |
| Congruency | -0.37 | 0.25 | -1.51 | 0.132 |
| Base rate | -0.40 | 0.34 | -1.16 | 0.256 |
| Critical word frequency | -0.12 | 0.15 | -0.80 | 0.423 |
| Congruency: Base rate | -0.14 | 0.49 | -0.29 | 0.774 |
|  |  |  |  |  |
| Models for openness analyses |  |  |  |  |
| N400 (300-500 ms) |  |  |  |  |
| Intercept | -1.74 | 0.17 | -10.52 | < .001 |
| Congruency | 0.03 | 0.26 | 0.11 | 0.910 |
| Base rate | -0.36 | 0.23 | -1.60 | 0.110 |
| Openness | 0.09 | 0.15 | 0.60 | 0.555 |
| Critical word frequency | 0.22 | 0.13 | 1.71 | 0.090 |
| Congruency: Base rate | -0.51 | 0.45 | -1.14 | 0.256 |
| Congruency: Openness | -0.01 | 0.23 | -0.03 | 0.978 |
| Base rate: Openness | 0.09 | 0.26 | 0.37 | 0.712 |
| Congruency: Base rate: Openness | -0.86 | 0.46 | -1.89 | 0.059 |
| P600 (600-1000 ms) |  |  |  |  |
| Intercept | 0.06 | 0.22 | 0.28 | 0.785 |
| Congruency | -0.37 | 0.25 | -1.51 | 0.132 |
| Base rate | -0.40 | 0.34 | -1.18 | 0.248 |
| Openness | 0.14 | 0.21 | 0.65 | 0.519 |
| Critical word frequency | -0.12 | 0.15 | -0.81 | 0.422 |
| Congruency: Base rate | -0.14 | 0.49 | -0.29 | 0.775 |
| Congruency: Openness | 0.03 | 0.25 | 0.11 | 0.915 |
| Base rate: Openness | -0.46 | 0.34 | -1.37 | 0.180 |
| Congruency: Base rate: Openness | -0.55 | 0.50 | -1.11 | 0.267 |
| Model for main analysis (300-500 ms): Amplitude ~ Congruency*Base rate + CWF + (1 \| Participant) + (Congruency + 1 \| Item); Model for main analysis (600-1000 ms): Amplitude ~ Congruency*Base rate + CWF + (Base rate + 1 \| Participant) + (1 \| Item); Model for openness analysis (300-500 ms): Amplitude ~ Congruency*Base rate*Openness + CWF + (1 \| Participant) + (Congruency + Base rate: Openness + 1 \| Item); Model for openness analysis (600-1000 ms): Amplitude ~ Congruency*Base rate*Openness + CWF + (Base rate + 1 \| Participant) + (1 \| Item) | | | | |

| **Table S4. LME models for TFR analyses in Experiment 1** | | | | |
| --- | --- | --- | --- | --- |
| Predictor | *β* | *SE* | *t* | *p* |
| Models for main analyses |  |  |  |  |
| High-beta power (21-30 Hz, 220-330 ms) |  |  |  |  |
| Intercept | 0.00 | 0.02 | 0.02 | 0.984 |
| Congruency | 0.00 | 0.03 | -0.07 | 0.943 |
| Base rate | -0.01 | 0.03 | -0.25 | 0.803 |
| Congruency: Base rate | 0.20 | 0.06 | 3.53 | < .001 |
| Theta power (4-6 Hz, 320-580 ms) |  |  |  |  |
| Intercept | 0.38 | 0.02 | 16.88 | < .001 |
| Congruency | 0.00 | 0.04 | 0.02 | 0.987 |
| Base rate | 0.03 | 0.05 | 0.71 | 0.480 |
| Congruency: Base rate | 0.24 | 0.08 | 3.01 | 0.003 |
|  |  |  |  |  |
| Models for openness analyses |  |  |  |  |
| High-beta power (21-30 Hz, 220-330 ms) |  |  |  |  |
| Intercept | 0.00 | 0.02 | 0.03 | 0.980 |
| Congruency | 0.00 | 0.03 | -0.12 | 0.908 |
| Base rate | -0.01 | 0.03 | -0.25 | 0.802 |
| Openness | 0.00 | 0.02 | 0.05 | 0.965 |
| Congruency: Base rate | 0.20 | 0.06 | 3.52 | < .001 |
| Congruency: Openness | -0.04 | 0.03 | -1.37 | 0.170 |
| Base rate: Openness | -0.02 | 0.03 | -0.55 | 0.589 |
| Congruency: Base rate: Openness | 0.03 | 0.06 | 0.47 | 0.637 |
| Theta power (4-6 Hz, 320-580 ms) |  |  |  |  |
| Intercept | 0.38 | 0.02 | 17.02 | < .001 |
| Congruency | 0.00 | 0.04 | 0.01 | 0.989 |
| Base rate | 0.03 | 0.05 | 0.71 | 0.481 |
| Openness | -0.02 | 0.02 | -1.11 | 0.277 |
| Congruency: Base rate | 0.24 | 0.08 | 3.01 | 0.003 |
| Congruency: Openness | -0.10 | 0.04 | -2.56 | 0.011 |
| Base rate: Openness | -0.06 | 0.04 | -1.53 | 0.126 |
| Congruency: Base rate: Openness | -0.04 | 0.08 | -0.48 | 0.632 |
| Model for main analysis (21-30 Hz): Power ~ Congruency*Base rate + (Congruency + Base rate + 1 \| Participant) + (1 \| Item); Model for main analysis (4-6 Hz): Power ~ Congruency*Base rate + (1 \| Participant) + (Base rate + 1 \| Item); Model for openness analysis (21-30 Hz): Power ~ Congruency*Base rate*Openness + (Base rate + 1 \| Participant) + (1 \| Item); Model for openness analysis (4-6 Hz): Power ~ Congruency*Base rate*Openness + (1 \| Participant) + (Base rate + 1 \| Item) | | | | |

| **Table S5. LME models for amplitude analyses in Experiment 2** | | | | |
| --- | --- | --- | --- | --- |
| Predictor | *β* | *SE* | *t* | *p* |
| Models for main analyses |  |  |  |  |
| N400 (300-500 ms) |  |  |  |  |
| Intercept | -1.41 | 0.19 | -7.43 | < .001 |
| Congruency | 0.04 | 0.25 | 0.17 | 0.862 |
| Base rate | 0.01 | 0.23 | 0.02 | 0.981 |
| Critical word frequency | 0.27 | 0.13 | 2.17 | 0.031 |
| Congruency: Base rate | 0.33 | 0.45 | 0.73 | 0.464 |
| P600 (600-1000 ms) |  |  |  |  |
| Intercept | 0.29 | 0.16 | 1.80 | 0.081 |
| Congruency | 0.27 | 0.24 | 1.15 | 0.249 |
| Base rate | 0.01 | 0.33 | 0.02 | 0.983 |
| Critical word frequency | -0.07 | 0.14 | -0.53 | 0.595 |
| Congruency: Base rate | -0.42 | 0.47 | -0.88 | 0.379 |
|  |  |  |  |  |
| Models for openness analyses |  |  |  |  |
| N400 (300-500 ms) |  |  |  |  |
| Intercept | -1.41 | 0.19 | -7.39 | < .001 |
| Congruency | 0.04 | 0.25 | 0.17 | 0.866 |
| Base rate | 0.01 | 0.23 | 0.03 | 0.975 |
| Openness | 0.15 | 0.18 | 0.81 | 0.425 |
| Critical word frequency | 0.28 | 0.13 | 2.20 | 0.030 |
| Congruency: Base rate | 0.33 | 0.45 | 0.73 | 0.465 |
| Congruency: Openness | -0.12 | 0.23 | -0.53 | 0.598 |
| Base rate: Openness | 0.18 | 0.23 | 0.78 | 0.437 |
| Congruency: Base rate: Openness | -0.55 | 0.46 | -1.20 | 0.232 |
| P600 (600-1000 ms) |  |  |  |  |
| Intercept | 0.29 | 0.16 | 1.76 | 0.087 |
| Congruency | 0.28 | 0.24 | 1.19 | 0.233 |
| Base rate | 0.02 | 0.32 | 0.05 | 0.959 |
| Openness | -0.07 | 0.15 | -0.45 | 0.656 |
| Critical word frequency | -0.09 | 0.14 | -0.67 | 0.503 |
| Congruency: Base rate | -0.42 | 0.47 | -0.89 | 0.375 |
| Congruency: Openness | -0.18 | 0.24 | -0.76 | 0.450 |
| Base rate: Openness | 0.34 | 0.33 | 1.06 | 0.299 |
| Congruency: Base rate: Openness | -0.08 | 0.47 | -0.17 | 0.862 |
| Model for main analysis (300-500 ms): Amplitude ~ Congruency*Base rate + CWF + (1 \| Participant) + (Congruency + 1 \| Item); Model for main analysis (600-1000 ms): Amplitude ~ Congruency*Base rate + CWF + (Base rate + 1 \| Participant) + (1 \| Item); Model for openness analysis (300-500 ms): Amplitude ~ Congruency*Base rate*Openness + CWF + (1 \| Participant) + (Congruency + 1 \| Item); Model for openness analysis (600-1000 ms): Amplitude ~ Congruency*Base rate*Openness + CWF + (Base rate + 1 \| Participant) + (Openness + 1 \| Item) | | | | |

| **Table S6. LME models for TFR analyses in Experiment 2** | | | | |
| --- | --- | --- | --- | --- |
| Predictor | *β* | *SE* | *t* | *p* |
| Models for main analyses |  |  |  |  |
| High-beta power (21-30 Hz, 340-390 ms) |  |  |  |  |
| Intercept | -0.03 | 0.02 | -1.47 | 0.152 |
| Congruency | -0.05 | 0.03 | -1.78 | 0.075 |
| Base rate | -0.06 | 0.03 | -1.93 | 0.054 |
| Congruency: Base rate | 0.16 | 0.06 | 2.71 | 0.007 |
|  |  |  |  |  |
| Models for openness analyses |  |  |  |  |
| High-beta power (21-30 Hz, 340-390 ms) |  |  |  |  |
| Intercept | -0.03 | 0.02 | -1.53 | 0.135 |
| Congruency | -0.05 | 0.03 | -1.82 | 0.069 |
| Base rate | -0.06 | 0.03 | -1.94 | 0.053 |
| Openness | -0.04 | 0.02 | -1.99 | 0.057 |
| Congruency: Base rate | 0.16 | 0.06 | 2.75 | 0.006 |
| Congruency: Openness | -0.04 | 0.03 | -1.30 | 0.195 |
| Base rate: Openness | 0.03 | 0.03 | 0.80 | 0.423 |
| Congruency: Base rate: Openness | 0.03 | 0.06 | 0.43 | 0.670 |
| Model for main analysis (21-30 Hz): Power ~ Congruency*Base rate + (1 \| Participant) + (1 \| Item); Model for openness analysis (21-30 Hz): Power ~ Congruency*Base rate*Openness + (1 \| Participant) + (Base rate: Openness + 1 \| Item) | | | | |
